# Incidence rates, causes and strategies to prevent hospital readmissions at National Hospital Galle, Sri Lanka

**DOI:** 10.1186/s12913-026-14972-7

**Published:** 2026-06-16

**Authors:** A. L. M. Payas, S. L. D. W. Abeyrathne, Thushari Bandara, G. H. S. Fernando, Ranga Sabapathige

**Affiliations:** 1 Department of Nursing, Faculty of Allied Health Sciences, University of Ruhuna, Galle, Sri Lanka; 2 Department of Medical Laboratory Science, Faculty of Allied Health Sciences, University of Ruhuna, Galle, Sri Lanka; 3 District General Hospital, Matale, Sri Lanka; 4 National Hospital Galle, Galle, Sri Lanka

**Keywords:** Incidence rate, Hospital readmission, Causative factors, Preventive strategies

## Abstract

**Background:**

A readmission is defined as an unplanned subsequent hospital admission in the same or a different hospital within 30 days after discharge from hospital due to the same illness. Readmission rates and its causative factors could be used to measure the quality of health care and accountability. Present study was undertaken to determine the incidence and causative factors of hospital readmissions at National Hospital Galle, Sri Lanka and to study the ongoing strategies for their prevention.

**Methods:**

This is a descriptive cross-sectional study. A pre-tested interviewer-administered questionnaire was used to collect data from re-admitted patients for consecutive seven days. Further data were collected from nursing officers (*n* = 102) who are participated in the patient discharge process at the same hospital to assess the strategies to prevent re-admissions. Data were analyzed using SPSS version 25.0, by using descriptive statistics, Pearson’s test and Chi-square tests wherever applicable.

**Results:**

This study assessed seven-day hospital readmissions among 4,492 patients admitted to the National Hospital Galle. A total of 946 patients were readmitted, during the study period giving an overall readmission rate of 21.06%. Most readmissions were planned (19.08%), while 1.98% were unplanned. Elderly patients (≥ 65 years) and those with multiple chronic conditions, including diabetes, hypertension, and heart failure, had higher readmission rates. Patients discharged within five days showed an increased risk of readmission. Readmitted patients reported lower satisfaction, particularly regarding communication and discharge instructions. Nursing staff surveys revealed dissatisfaction with discharge processes, inadequate patient education, limited post-discharge follow-up, and insufficient time and training for discharge counselling.

**Conclusions:**

Findings concluded that National Hospital Galle experienced a notable rate of general readmissions, while unplanned readmissions remained low. These return visits were heavily driven by patient dissatisfaction with post-discharge support and discharge communication. Consequently, this highlights a critical need to enhance patient education and follow-up protocols.

**Supplementary Information:**

The online version contains supplementary material available at 10.1186/s12913-026-14972-7.

## Background

Hospital readmission, commonly defined as an unplanned admission to the same or a different hospital within 30 days following discharge for the same condition, is widely recognized as an important indicator of healthcare quality and continuity of care [1]. High readmission rates are associated with increased healthcare costs, overcrowding of hospitals, and adverse patient outcomes, including physical, psychological, and social burdens [2]. Consequently, reducing avoidable hospital readmissions has become a priority for healthcare systems worldwide.

International evidence indicates that a significant proportion of readmissions are preventable and often result from inadequate discharge planning, poor patient education, limited follow-up care, medication non-adherence, and socio-economic constraints [3, 4]. Elderly patients and individuals with chronic diseases such as diabetes, cardiovascular disease, chronic obstructive pulmonary disease, and mental health disorders are particularly vulnerable to repeated hospitalizations [5, 6]. Short hospital stays and insufficient post-discharge support further increase the risk of unplanned readmissions [7].

Nurses play a pivotal role in preventing hospital readmissions through comprehensive discharge planning, patient and family education, medication reconciliation, and coordination of post-discharge care [8]. Evidence suggests that nurse-led interventions, including structured discharge protocols, follow-up calls, and community-based support, can significantly reduce readmission rates and improve patient outcomes [9, 10]. However, the effectiveness of these strategies depends on institutional support, availability of resources, and consistent implementation.

Despite extensive international research, there is a notable lack of empirical evidence on hospital readmission rates, associated factors, and preventive strategies within the Sri Lankan healthcare context. Limited local studies highlight gaps in discharge education, follow-up mechanisms, and community support systems [11]. Understanding the incidence, causes, and existing preventive practices of hospital readmissions at the National Hospital Galle is therefore essential to inform targeted interventions, improve quality of care, and support evidence-based policy development.

## Methods

A descriptive cross-sectional study design was utilized to comprehensively evaluate the incidence of hospital readmissions, examine patient- and hospital-related contributing factors, and assess strategies aimed at preventing readmissions at the National Hospital Galle located in Southern Sri Lanka. This is the third largest tertiary care center in Sri Lanka, delivers specialized medical, surgical, pediatric, and psychiatric services and functions as a major referral center for the region. The study was conducted from April 30 to 07 May in 2025. Admission and readmission patterns during the selected week were compared with corresponding hospital records from the previous year to ensure that the patient volume and service utilization were broadly consistent with typical hospital activity. The population of the study was the patients who were admitted more than one time within 30 days for same disease condition to National Hospital Galle and nursing officers who working in medical wards, surgical wards, pediatrics wards and psychiatric wards involving in discharge procedure in National Hospital Galle.

The study population consisted of two main groups: patients who experienced unplanned readmission within 30 days following discharge for the same clinical condition and nursing officers who were directly involved in discharge planning, patient education, and continuity of care are eligible for this study. Patients admitted for oncology and dialysis units were excluded because their readmissions are typically scheduled as part of long-term treatment protocols. As well as Nurses who were leave on during study period and nurses who were not willing to participate in the study were excluded from this study.

First part of the study was conducted using purposive sampling technique. Data collection was conducted over a continuous seven-day period, during which all eligible patients meeting the inclusion criteria were recruited through consecutive sampling method and a total of 89 unplanned readmission cases were found.

Sample size for the nursing officers who involved in the second part of the study was calculated using the sample size calculation for finite population formula by Lawanga and Lemeshow, 1991, as follows.

$$\:=\:\frac{{\mathrm{z}}^{2}\mathrm{p}\mathrm{q}\mathrm{N}}{{\mathrm{e}}^{2}\left(\mathrm{N}-1\right)+{\mathrm{z}}^{2}\mathrm{p}\mathrm{q}}$$

$$\:n=\:\frac{{1.96}^{2}*0.08*(1-0.08)1000}{{0.05}^{2}\left(1000-1\right)+{1.96}^{2}0.08*(1-0.08)}$$

$$\:n=\:\frac{282.74176}{2.78024}$$

$$\:n=101.69\:$$

n = required sample size, z = Critical value of specified confidence = 1.96, p = Probable estimate of proportions of characteristics of interest = 0.08 [12], q = 1-p, N = Size of the total population. According to the hospital = 1000 (Data from National Hospital Galle).

According to the calculation, the minimum sample size of nursing officers was taken as 102.

A stratified random sampling technique was employed to ensure proportional representation of nursing officers from different wards and clinical units within the hospital.

Data were collected using two structured, pre-tested questionnaires specifically developed for this study, by the investigators. An interviewer-administered questionnaire was used for patients and comprised 57 items designed to gather detailed information on socio-demographic characteristics, medical history, comorbid conditions, previous hospitalizations, discharge planning experiences, medication instructions, follow-up arrangements, and circumstances surrounding readmission. A self-administered questionnaire was used for nursing officers and included 61 items across ten domains addressing discharge planning practices, patient education, medication reconciliation, coordination of multidisciplinary care, communication methods, and strategies implemented to reduce preventable readmissions. The development of both questionnaires was guided by an extensive review of relevant literature and clinical guidelines and was made available in Sinhala, Tamil, and English to ensure participant comprehension.

Eligible readmitted patients were identified on a daily basis using the hospital information management system and admission records. To estimate the annual and monthly readmission burden, the observed readmission rates obtained during the study period were applied to the total number of hospital admissions recorded at National Hospital Galle during 2024 (*n* = 204,164). Monthly estimates were calculated by dividing annual estimates by 12. This approach was used instead of direct weekly extrapolation to provide a more representative estimate of the annual readmission burden. Interviews were conducted in the respective wards at times that were convenient for the patients and did not interfere with clinical care. Nursing officers were approached during duty hours, provided with information regarding the study objectives, and invited to participate. Questionnaires were distributed after obtaining written informed consent and were collected either on the same day or within an agreed period to minimize disruption to routine duties. To ensure the quality, reliability, and clarity of the data collection tools, a pre-test was conducted among ten patients and ten nursing officers in a comparable healthcare setting. Feedback obtained during the pre-testing phase was used to refine question wording, structure, and sequencing. In all statistical tests *p* values < 0.05 was considered as statistically significant.

### Results of the 1st part

The overall 30-day readmission incidence rate was 21.06% (946 patients). This includes 19.08% (857 patients) of planned readmissions and 1.98% (89 patients) unplanned readmissions. These rates correspond to approximately, 21 planned readmissions per 100 patients weekly or 210 per 1,000 patients weekly and 2 unplanned readmissions per 100 patients weekly or 20 per 1,000 patients weekly. Summary of readmission incidence rate was indicated in Table 1.

**Table 1:** Summary of readmission data (one-week study period) (*n* = 4492)

Category	Number of patients	Percentage of total (*n* = 4492)
Total admissions assessed	4,492	100%
Admissions not within 30 days	3,417	76.07%
Initial 30-day admissions identified	1,075	23.93%
Confirmed readmissions – Planned readmissions – Unplanned readmissions	946	21.06%
	857	19.08%
	89	1.98%
Non-readmissions (excluded)	120	2.67%
Untraceable patients	9	0.20%

Based on the observed readmission rates and the total number of hospital admissions recorded at National Hospital Galle in 2024 (*n* = 204 164), the estimated annual readmission burden was 42,996 (204164*21.06/100) patients, including 38,954 (204164*19.08/100) planned readmissions and 4,042 (204164*1.98/100) unplanned readmissions. The corresponding monthly estimates were 3,583 (42996/12) total readmissions, comprising 3,246 (38954/12) planned and 337 (4042/12) unplanned readmissions. Based on the average weekly admission volume in 2024, the estimated weekly readmission burden was 827 (42996/52) patients, including 749 (38954/52) planned and 78 (4042/52) unplanned readmissions. A comparison with the study week showed that 89 unplanned readmissions were observed, which was comparable to the estimated weekly figure. The estimated monthly, annual, and weekly readmission projections are presented in Table 2.

**Table 2:** Estimated annual and monthly readmission burden based on 2024 hospital admissions (*n* = 204,164)

Readmission Type	Observed Rate (%)	Annual Estimate	Monthly Estimate	Weekly Incidence
Planned	19.08	38,954	3,246	749
Unplanned	1.98	4042	337	78
Total Readmissions	21.06	42,996	3583	827

The present study involved a group of 89 patients. The gender distribution showed a higher proportion of males (55.1%) than females (44.9%). Age distribution skewed towards the elderly, with 38.2% of patients aged over 60 years and only 13.5% below 20 years. This aligns with literature indicating that older adults are more prone to readmissions due to co morbidities and functional limitations. A substantial majority of patients were married (76.4%) and Sinhala (93.3%), reflecting regional population patterns. The predominant religion was Buddhism (89.9%). Educational attainment was relatively low, with 15.7% never attending school and 38.2% having only primary-level education. Notably, 59.6% of participants were unemployed, and 39.3% had no source of income, suggesting that socioeconomic vulnerability may play a role in post-discharge challenges. To better understand the social demographic characteristics of these patients, their information was compiled and presented in Table 3.

**Table 3:** Socio-demographic characteristics of the re-admitted patients (*n* = 89)

		Count	Column N %
Gender	Female	40	44.9%
	Male	49	55.1%
Age group	< 20	12	13.5%
	20–30	6	6.7%
	31–40	10	11.2%
	41–50	11	12.4%
	51–60	16	18.0%
	> 60	34	38.2%
Marital status	Married	68	76.4%
	Unmarried	19	21.3%
	Separated	0	0.0%
	Divorced	0	0.0%
	Widowed	2	2.2%
Nationality	Sinhala	83	93.3%
	Muslim	3	3.4%
	Tamil	3	3.4%
	Burgher	0	0.0%
	Other	0	0.0%
Religion	Buddhism	80	89.9%
	Christianity	3	3.4%
	Islamic	3	3.4%
	Hindu	3	3.4%
	Other	0	0.0%
Educational status	Not schooling	14	15.7%
	Grade 5	34	38.2%
	O/L	14	15.7%
	O/L Pass	15	16.9%
	A/L	7	7.9%
	A/L Pass	4	4.5%
	Graduate	1	1.1%
Employment status	Yes	36	40.4%
	No	53	59.6%
Monthly income	No income	35	39.3%
	< 10,000	12	13.5%
	10,000–20,000	2	2.2%
	20,000–50,000	31	34.8%
	50,000–100,000	9	10.1%
	> 100,000	0	0.0%

Table 4 summarizes patient-related factors associated with unplanned readmissions (*n* = 89). A majority of patients reported very good understanding of discharge instructions (67.4%) and health education (66.3%), and a similar proportion took medications without missing doses (67.4%). Follow-up-related challenges were reported by 52.8% of patients. Although most patients demonstrated concern about their health (84.3%), adequate home support (94.4%), and preparedness for home care (89.9%), adherence to nutrition education was relatively low, with only 22.5% fully following recommended dietary advice. Overall, the findings suggest that while general discharge understanding and support systems were strong, gaps in follow-up and nutritional adherence may have contributed to readmissions.

**Table 4:** Patients related factors for readmissions

Patient-Related factors	Number of patients (*n* = 89)	Percentages (%)
Understood discharge (Very Well)	60	67.4
Follow-up issues (Yes)	47	52.8
Took medicines without missing	60	67.4
Understood health education (Very Well)	59	66.3
Followed health education (Yes)	55	61.8
Understood nutrition education (Very Well)	40	44.9
Followed nutrition (Fully)	20	22.5
Prepared for home care	80	89.9
Adequate home support	84	94.4
Able to follow-up (always + most)	71 (30 + 41)	79.8
Concern about health (Yes)	75	84.3

Table 5 shows how hospital care and discharge processes were experienced by the 89 patients who were readmitted. Most patients felt that the basic steps were done properly before discharge. Almost all patients were given discharge instructions (91%) and information about their medications (92.1%). A large proportion were also told to attend follow-up clinics (85.4%) and received some form of health education (78.7%) and nutrition advice (74.2%). This indicates that routine discharge communication was generally provided.

Despite this, important gaps were identified in areas that directly support patients after going home. Only a small number of patients (18%) received a written care plan, which means many had to rely on memory or verbal instructions alone. In addition, less than half of the patients (43.8%) felt that their follow-up care was very satisfactory, suggesting problems with continuity of care after discharge. Teaching on how to manage medical devices was particularly poor, with only 9% receiving such instructions, placing these patients at higher risk of complications once they returned home.

Most patients (77.5%) stayed in hospital for only 1–3 days. While short hospital stays are often necessary, they may reduce the time available for detailed patient education and discharge planning. Although over half of the patients described their overall hospital experience as excellent (53.9%) and many felt well supported by the healthcare team (76.4%), these positive experiences did not always lead to smooth recovery after discharge.

**Table 5:** Hospital related factors for readmissions

Hospital related factors	Number of patients (*n* = 89)	Percentages (%)
Received discharge instructions	81	91
Told to attend follow-up	76	85.4
Instructed about medications	82	92.1
Instructed about health education	70	78.7
Instructed about nutrition	66	74.2
Instructed to manage devices	8	9
Hospital stay 1–3 days	69	77.5
Overall Hospital Experience (Excellent)	48	53.9
Support from healthcare team (very supported)	68	76.4
Received written care plan	16	18
Follow-up care very satisfactory	39	43.8

### Results of the 2nd Part

Table 6 presents the socio-demographic characteristics of the nurses who participated in the study (*n* = 102). The mean age of the respondents was 38 years, with an age range of 28 years and a standard deviation of 7, indicating a predominantly middle-aged nursing workforce. The sample was largely female, with 95 nurses (93.1%), while only 7 participants (6.9%) were male, reflecting the female predominance commonly observed in the nursing profession.

With regard to ethnicity, the majority of respondents were Sinhalese (98.0%, *n* = 100), whereas a small proportion belonged to Christianity (2.0%, *n* = 2). In terms of professional experience, nurses had a mean of 12 years of service, with a wide range of experience spanning up to 34 years and a standard deviation of 7, suggesting a mix of moderately experienced and highly experienced nurses in the study setting.

Concerning professional position, most participants were Nursing Officers (79.4%, *n* = 81). This was followed by Nursing Officer Grade I (15.7%, *n* = 16). A smaller number of respondents held senior positions, including Nursing Officer Grade II (2.0%, *n* = 2), Nursing Officer Grade III (2.0%, *n* = 2), and Nursing Sister (1.0%, *n* = 1). Overall, the table indicates that the study sample consisted mainly of experienced female nursing officers, providing a relevant perspective on hospital practices related to patient care and readmission prevention.

**Table 6:** Socio-demographic characteristics of the nursing officers (n = 102)

		Count	Column N %	Mean	Range	Standard Deviation
Age		102	100.00%	38	28	7
Gender	Female	95	93.10%			
	Male	7	6.90%			
Ethnicity	Christianity	2	2.00%			
	Sinhalese	100	98.00%			
Experience years		102	100.00%	12	34	7
Position	Nursing officer	81	79.40%			
	Nursing officer-Grade I	16	15.70%			
	Nursing officer-Grade II	2	2.00%			
	Nursing officer -Grade III	2	2.00%			
	Nursing Sister	1	1.00%			

Table 7 presents the strategies currently used by nurses to prevent hospital readmissions. The findings show that nurses are actively involved in multiple preventive practices across readmission tracking, discharge planning, medication management, patient education, follow-up care, training, and patient engagement. Regarding readmission tracking, the most commonly used method was manual tracking, reported by 51 nurses (50.0%), followed by electronic health record (EHR) tracking used by 33 nurses (32.4%). In contrast, the use of standard protocols (10.8%), informal or personal methods (6.9%), and diagnosis cards or clinic books (1.0%) was relatively low, indicating limited reliance on structured or standardized tracking systems.

In terms of discharge planning, patient education at discharge was the most frequently practiced strategy, reported by 71 nurses (69.6%). Planning for high-risk patients was also commonly implemented (52.9%), while discharge checklist use was reported by 38.2% of nurses. These findings highlight the emphasis placed on education-based discharge processes, though checklist-based approaches were less consistently applied. With respect to medication management, double-checking medications was the most widely used strategy (60.8%), followed by medication reconciliation (56.9%) and clarifying medication instructions (53.9%). This suggests a strong focus on medication safety practices aimed at reducing medication-related readmissions.

Concerning patient education, verbal education was provided by 59.8% of nurses, while nutrition and lifestyle education was reported by 51.0%. Written educational materials were less frequently used (45.1%), indicating a greater reliance on verbal communication rather than written resources. In the area of follow-up care, only 26.5% of nurses reported arranging post-discharge phone calls, and home visits after discharge were reported by just 8.8% of nurses. This demonstrates limited post-discharge follow-up practices, which may contribute to higher readmission risk.

Regarding training and support, lack of training was reported by a substantial proportion of nurses (60.8%), while only 38.2% indicated that they had received training related to readmission prevention, suggesting a notable gap in professional development. Finally, under patient engagement and communication, patient involvement in discharge planning was reported by 47.1% of nurses. Collaboration with family members (44.1%) and communication with caregivers (46.1%) were moderately practiced, indicating partial but inconsistent engagement of support systems in the discharge process.

**Table 7:** – Currently use strategies to prevent hospital readmissions

Category	Preventive strategies	Number of nurses		Percentages (%)
Readmission tracking	Manual tracking	51	50	
	EHR tracking	33	32.4	
	Standard protocols	11	10.8	
	Informal/personal methods	7	6.9	
	Diagnosis card/clinic books	1	1	
Discharge planning	Patient education on discharge	71	69.6	
	Planning for high-risk patients	54	52.9	
	Discharge checklist use	39	38.2	
Medication management	Double-check medications	62	60.8	
	Medication reconciliation	58	56.9	
	Clarify medication instruction	55	53.9	
Patient education	Verbal education	61	59.8	
	Written materials	46	45.1	
	Nutrition/lifestyle education	52	51	
Follow-up care	Post-discharge phone calls	27	26.5	
	Home visits after discharge	9	8.8	
Training & support	Received training	39	38.2	
	Felt lack of training	62	60.8	
Patient engagement & communication	Patient involvement in discharge	48	47.1	
	Collaboration with family	45	44.1	
	Communication with caregivers	47	46.1	

For this study only readmitted patients were included, and a comparison group of non-readmitted patients was not available. Therefore, it was not methodologically appropriate to calculate p-values or odds ratios for associations with readmission. However, we statistically analysed some association between two factors.

#### Association between monthly income and access to follow-up care

The relationship between monthly income and issues obtaining follow-up care after discharge was investigated using the Chi-square analysis. Of the 89 respondents, 47.2% (*n* = 42) did not report follow-up problems, whereas 52.8% (*n* = 47) did. The majority of patients with no income (*n* = 35) 62.9% and (*n* = 22) reported having trouble getting follow-up services. In a similar vein, 66.7% (*n* = 8) of people making less than LKR 10,000 experienced problems. By comparison, only 38.7% (*n* = 12) of patients who made between LKR 20,000 and 50,000 and 33.3% (*n* = 3) of those who made between LKR 50,000 and 100,000 reported having such issues. Notably, access problems affected 100% (*n* = 2) of people making between LKR 10,000 and 20,000. While there was a discernible downward trend in difficulty as income increased, there was no statistically significant correlation, according to the Chi-square test result (*p* = 0.093). Nonetheless, the pattern implies that low-income patients might encounter more obstacles to receiving ongoing care, possibly as a result of lack of support, transportation problems, or financial limitations.

#### Educational level and understanding of discharge-related instructions

Multiple Chi-square tests were used to assess the relationship between educational status and comprehension of instructions related to discharge. Of all patients, 67.4% (*n* = 60) said they understood the discharge instructions “very well.” Of them, 16.4% (*n* = 10) had completed O/L coursework, and 37.7% (*n* = 23) had completed Grade 5. It’s interesting to note that 14.8% (*n* = 9) of these patients had no formal education, suggesting that comprehension was not completely impeded by low literacy. Discharge understanding and educational attainment did not significantly correlate (*p* = 0.906). About 64.0% (*n* = 57) of respondents said they understood the medication instructions “very well,” and once more, the most common levels were Grade 5 (38.6%, *n* = 22) and O/L (19.3%, *n* = 11). However, this association was likewise not statistically significant (*p* = 0.620). Similarly, 66.3% (*n* = 59) said they understood health education (*p* = 0.751), and 44.9% (*n* = 40) said they understood nutrition advice (*p* = 0.801). Regardless of educational attainment, the majority—91.0% (*n* = 81)—answered “not at all” when asked if they understood how to manage medical devices, while only 5.6% (*n* = 5) said they understood “very well.” Additionally, this association (*p* = 0.619) was not statistically significant. These findings demonstrate that discharge comprehension is not solely determined by educational attainment. Even patients with little formal education may benefit from hospital staff using effective communication techniques to close gaps.

## Discussion

The findings revealed that the overall 30-day readmission rate was 21.06%, of which 19.08% were planned readmissions and only 1.98% were unplanned readmissions. This indicates that more than 90% of all readmissions were planned, highlighting a distinctive readmission pattern at NHG when compared with international healthcare settings [1, 2]. Based on 2024 admission data, the estimated average weekly number of unplanned readmissions was 78 patients, compared with 89 observed during the study week. This close agreement suggests that the observed readmission rate is a reasonable estimate of the hospital’s unplanned readmission burden. Globally, reported 30-day readmission rates vary considerably. A large systematic review conducted in China reported an overall readmission rate of 7.6%, including 6.7% planned and 0.9% unplanned readmissions [3]. In contrast, studies from Hong Kong and Australia have reported unplanned readmission rates of 16.7% and 7.4%, respectively [4, 5]. While the total readmission rate at NHG (21.06%) is comparable to rates reported from other tertiary hospitals, the proportion of planned readmissions (19.08%) is markedly higher. This finding may be explained by the hospital’s role as a tertiary referral center, where patients frequently require repeated admissions for scheduled procedures, long-term therapies, and follow-up interventions [6].

The unplanned readmission rate of 1.98% observed in this study is substantially lower than the 5–20% range reported internationally [7, 8]. However, this low rate should be interpreted with caution. Oncology and dialysis units were excluded from the study, despite these units accounting for a significant proportion of unplanned readmissions in other settings. In addition, the short study period may have contributed to underestimation. Therefore, the low unplanned readmission rate may reflect methodological limitations rather than superior quality of care [2, 7].

Patient-related factors were found to play an important role in readmissions. The elderly population accounted for a higher proportion of readmitted patients, particularly those aged 65 years and above, consistent with global evidence that aging populations are at increased risk of hospital readmission [5, 9, 10]. Furthermore, patients with multiple chronic conditions such as diabetes, hypertension, and heart failure showed a significantly higher likelihood of readmission [11]. Shorter hospital stays were also associated with increased readmission risk, with patients discharged within five days more frequently returning to hospital, suggesting possible premature discharge or inadequate stabilization [13].

Discharge-related factors emerged as major contributors to readmissions. Although patients reported overall satisfaction with inpatient care, only 18% of patients received a written discharge care plan, indicating a substantial gap in structured discharge communication [14]. Additionally, 30% of patients did not adhere to dietary recommendations after discharge, highlighting deficiencies in patient understanding and follow-through [14, 15]. These findings align with international literature identifying poor discharge education and inadequate communication as major drivers of preventable readmissions [6, 16, 17].

The nursing officers’ perspective further reinforced these findings. More than two-thirds of nurses reported dissatisfaction with current discharge processes, and only a minority confirmed routine post-discharge follow-up such as phone calls or home visits [18, 19]. Medication reconciliation was reported as always performed by only 41.0% of nurses, while 34.4% indicated it was done only sometimes, increasing the risk of medication errors after discharge [20]. Additionally, only 29.5% of nurses reported regular involvement of a multidisciplinary team in discharge planning, and just 16.4% confirmed the use of electronic systems to track discharged patients or coordinate follow-up care [21, 22].

## Limitations of the study

This research was carried out in only one hospital (National Hospital Galle) therefore the results may not necessarily indicative of what is going on generally in other hospitals in Sri Lanka. Information collected through self-administered questionnaire may carry recall bias. Further, as information was gathered within a short time window, so it may not reflect seasonal or festival-related variation in readmission trends. Data collection was conducted over a single seven-day period; therefore, the findings may not fully reflect week-to-week variations in readmission patterns. The study did not assess the role of intern medical officers in the discharge process; therefore, their potential influence on hospital readmission outcomes could not be evaluated. However, it is also important to note that in our hospital context, nurses play an equally significant role in discharge planning and patient education. Nursing staff are actively involved in providing discharge instructions, counseling patients, and ensuring continuity of care.

## Conclusions and recommendations

This study evaluated readmission dynamics at the National Hospital Galle, examining hospital- and patient-level drivers alongside current preventive strategies. The findings indicate that while unplanned readmissions remain within expected bounds, planned readmissions constitute a notable portion of hospital utilization, underscoring readmission as a persistent challenge requiring targeted intervention.

On the patient side, older age, multimorbidity, and lower satisfaction with care emerged as key characteristics linked to readmission. Conversely, systemic hospital gaps were identified in discharge planning, transitional care coordination, and multidisciplinary teamwork. While discharge instruction delivery is widely practiced by nursing staff, structured post-discharge follow-up remains critically underutilized. Furthermore, essential clinical safeguarding mechanisms—specifically medication reconciliation and integrated digital health systems—are inconsistently applied. When compared to international benchmarks, the institution successfully implements several standard practices but falls short in leveraging continuous post-discharge communication, pharmacist-led interventions, and electronic health records. We recommend future studies should assess the role of intern medical officers in discharge planning, patient education, medication reconciliation, and follow-up coordination to better understand their influence on hospital readmission outcomes.

In conclusion, this study demonstrates that optimizing discharge preparedness and strengthening the continuity of transitional care are pivotal to reducing avoidable readmissions. Implementing these systematic refinements offers a practical pathway for healthcare facilities to enhance patient outcomes, streamline care delivery, and alleviate operational strain on the health system.

## Supplementary Information

Below is the link to the electronic supplementary material.

Supplementary Material 1

## Data Availability

The data that support the findings of this study are available from the corresponding author, upon reasonable request. All the data obtained in the current study were available from the corresponding authors on reasonable request.

## Abbreviations

AMI: Acute Myocardial Infarction
COPD: Chronic Obstructive Pulmonary Disease
CVD: Cardiovascular disease
HBSNFs: Hospital-based Skilled Nursing Facilities
HRRP: Hospital Readmission Reduction Program
LTDs: Long-Term Chronic Diseases
NHG: National Hospital Galle
NIMH: National Institute of Mental Health
PCPs: Primary Care Physician
SSHs: Short-Stay Hospitalizations
UK: United Kingdom
USA: United States of America
