# Supplementary material for: Incidence rates, causes and strategies to prevent hospital readmissions at National Hospital Galle, Sri Lanka
Source: BMC Health Serv Res. 2026 Jun 16;26:1189. doi: 10.1186/s12913-026-14972-7 (PMC13508404; doi:10.1186/s12913-026-14972-7)
Supplement: Supplementary file 1 [file 12913_2026_14972_MOESM1_ESM.pdf]

Questionnaire for patients – English format

Incidence rates, Causes and Strategies to Prevent Hospital Readmissions at National Hospital Galle

Date: Serial No:

Section 1 – Socio demographic data

Please marked with a right symbol for applicable answer (√)

1. Name: …………………………………………… (for identification purpose only)

2. Gender I. Female ☐ II. Male ☐

3. Age in years I. <20 years ☐ II. 21-30 years ☐

III. 31-40 years ☐ IV. 41- 50 years ☐

V. 51 - 60 years ☐ VI. >60 years ☐

4. Marital status I. Married ☐ II. Unmarried ☐

III. Separated ☐ IV. Divorced ☐

V. Widowed ☐

5. Nationality I. Sinhala ☐ II. Muslim ☐

III. Tamil ☐ IV. Burgher ☐

V. Other ☐

6. Religion I. Buddhism ☐ II. Christianity ☐

III. Islamic ☐ IV. Hindu ☐

V. Other ☐

7. Educational status

I. Not schooling ☐ II. Up to grade 5 ☐

III. Up to G.C.E.O/L ☐ IV. G.C.E. O/L pass ☐

V. Up to G.C.E. A/L ☐ VI. G.C.E.A/L pass ☐

VII. Graduate ☐

8. Are you employed? I. Yes ☐ II. No ☐

9. Monthly income (LKR)?

I. <10 000 ☐II. 10 000-20 000 ☐

III. 20 000-50 000 ☐IV. 50 000-100 000 ☐

V. >100 000 ☐

10. Do you admit to a hospital within 30 days for same condition?

I. Yes ☐ II. No ☐

Section 2- Health and hospitalization history

11. Previous admission Date……………..

12. How many times have you been hospitalized in the past month?

1. 1 ☐
2. 2 ☐
3. 3 ☐
4. 4 ☐

13. What was the on-admission complaint

1. Pain ☐
2. Fever ☐
3. Accident ☐
4. Other ☐

14. If other mention the complaint: ....

15. What was the previous diagnosis of admission? ....

16. How long was your last hospital stay?

1. 1-3 days ☐

2. 4-7 days ☐

3. 7-10 days ☐

4. More than 7 days ☐

17. Discharged Date: ....

18. Discharge type

1. Planned discharge ☐

2. LAMA - Leave again medical advices ☐

3. Leave from hospital without proper discharge ☐

4. Others.................................

Section 3- Experiences and perceptions

19. Did you get discharge instructions?

1. Yes ☐

2. No ☐

20. If yes, how well did you understand your discharge instructions?

1. Very well ☐

2. Somewhat ☐

3. Not very well ☐

4. Not at all ☐

21. Did you instructed to attend follow-up?

1. Yes ☐

2. No ☐

22. Have you experienced any issues with accessing follow-up after discharge (e.g., transportation, financial constraints)?

1. Yes (please specify): ....

2. No ☐

23. How satisfied were you with the follow-up care provided?

1. Very satisfied ☐

2. Satisfied ☐

3. Neutral ☐

4. Dissatisfied ☐

5. Very dissatisfied ☐

24. How many medications were you prescribed upon discharge?

1. 0-2 ☐

2. 3-4 ☐

3. 5-10 ☐

4. More than 10 ☐

25. Did you instruct about medications?

1. Yes ☐

2. No ☐

26. If yes, how well did you understand your medication instructions?

1. Very well ☐

2. Somewhat ☐

3. Not very well ☐

4. Not at all ☐

27. Did you take medication without miss?

1. Yes ☐

2. No ☐

28. If no how many times missed?

1. 1-3 ☐

2. 3-5 ☐

3. 5-7 ☐

4. 7-10 ☐

29. Reason for miss?

1. Expensive ☐

2. Don't like to take medication ☐

3. Forgot ☐

4. Other..................................

30. Did you instruct about health education during discharge?

1. Yes ☐

2. No ☐

31. If yes, how well did you understand your health education?

1. Very well ☐

2. Somewhat ☐

3. Not very well ☐

4. Not at all ☐

32. Did you follow the lhealth education?

1. Yes ☐

2. Some ☐

3. No ☐

33. If no or somewhat is the reason for miss?

1. It was very hard ☐

2. I don't like to follow health education ☐

3. I forgot ☐

4. Other..

34. Did you get instructions about Nutritional education?

1. Yes ☐

2. No ☐

35. If yes, how well did you understand your Nutritional education?

1. Very well ☐

2. Somewhat ☐

3. Not very well ☐

4. Not at all ☐

36. Did you take all nutrition according to you instructed?

1. Yes ☐

2. Some ☐

3. No ☐

37. If no or somewhat is reason for miss

1. Those are expensive ☐

2. I don't like ☐

3. I forgot ☐

4. Others................................

38. Discharged with any of the following (check all that apply):

1. Nasogastric tube ☐

2. Foley catheter ☐

3. Trachea tube ☐

4. Other indwelling catheter..........................

39. Did you instructed to how to manage above mentioned instruments

1. Yes ☐

2. No ☐

40. If yes, how well you understand the instructions?

1. Very well ☐

2. Somewhat ☐

3. Not very well ☐

4. Not at all ☐

41. Did you follow instructions?

1. Yes ☐

2. No ☐

42. If no, why not follow the instructions

1. It was very difficult to me ☐

2. I don't understand to handle instruments ☐

3. I forgot ☐

4. Others..............................

43. Did you feel involved in the decision-making process about your treatment?

1. Yes, very involved ☐

2. Somewhat involved ☐

3. Not very involved ☐

4. Not at all involved ☐

44. How would you rate your overall experience with the hospital care you received?

1. Excellent ☐

2. Good ☐

3. Fair ☐

4. Poor ☐

45. Did you feel supported by the healthcare team during your hospital stay?

1. Yes, very supported ☐
2. Somewhat supported ☐
3. Not very supported ☐
4. Not at all supported ☐

46. After being discharged, did you feel prepared to take care of yourself at home?

1. Yes, very prepared ☐
2. Somewhat prepared ☐
3. Not very prepared ☐
4. Not at all prepared ☐

47. Did you receive a written care plan upon discharge?

1. Yes ☐
2. No ☐

Section 4- Post-discharge support

48. Did you have adequate support at home after discharge?

1. Yes ☐
2. No ☐

49. Were you able to follow up on your medical appointments and medication regimen?

1. Yes, always ☐
2. Most of the time ☐
3. Sometimes ☐
4. Rarely ☐
5. Never ☐

50. Did you feel the need to be readmitted due to inadequate recovery or complications?

a. Yes ☐

b. No ☐

Section 5- Emotional and psychological impact

51. How often did you experience negative emotions (e.g., frustration, hopelessness) related to your health condition after discharge?

1. Very often ☐
2. Often ☐
3. Sometimes ☐
4. Rarely ☐
5. Never ☐

52. Did you feel like a burden to your family or healthcare providers during your hospital stay?

1. Yes, very much ☐
2. Somewhat ☐
3. Not really ☐
4. Not at all ☐

53. Did you have any concerns about your ability to manage your health after discharge?

1. Yes ☐
2. No ☐

Section 6- Readmission details

54. Have you been readmitted to the hospital within 30 days of your last discharge?

1. Yes ☐
2. No ☐

55. If yes, what was the reason for your readmission?

…………………………………………….

56. Was your readmission related to a previously diagnosed condition?

1. Yes ☐

2. No ☐

57. Did you experience any complications post-discharge that led to readmission?

1. Yes ☐

2. No ☐

Questionnaire for nursing officers/nursing In charges/nursing sisters – English format

Incidence rates, causes and Strategies to Prevent Hospital Readmissions at National Hospital Galle.

Date: Serial No:

Please underline the applicable answer

Section 1- Demographic data

1) Age: ....

2) Gender……………………….

3) Ethnicity: ....

4) Ward/unit: ....

5) Experience years: ....

6) Position: ....

Section 2: Understanding of hospital readmissions

7) How familiar are you with the concept of hospital readmissions?

a. Very familiar

b. Somewhat familiar

c. Not familiar at all

8) What do you consider the primary reasons for patient readmissions?

a. Medication non-adherence

b. Complications related to the initial condition

c. Inadequate follow-up care

d. Patient's social or economic factors

e. Other (please specify)...

9) How does your unit define and track hospital readmissions?

a. Standardized criteria and protocols

b. Electronic health record (EHR) tracking

c. Manual tracking through patient follow-ups

d. Other (please specify).......................................

10) What impact do you think readmissions have on patient outcomes and healthcare costs?

a. Significant negative impact

b. Moderate negative impact

c. Minimal impact

d. Positive impact

e. Unsure

Section 3: Discharge planning:

11) How frequently do you participate in discharge planning for patients?

a. Very frequently

b. Rarely

c. Never

12) How do you prioritize patients for comprehensive discharge planning?

a. Based on clinical complexity

b. High-risk patient criteria (e.g., previous readmissions)

c. Length of hospital stay

d. Other (please specify): ....

13) How do you ensure continuity of care during the discharge planning process?

a. Communication with primary care providers

b. Transfer of medical records and care plans

c. Educating patients about post-discharge care responsibilities

d. Other (please specify): ....

14) How do you involve patients and their families in the discharge planning process?

a. Educating them about the importance of follow-up care

b. Encouraging questions and concerns

c. Providing written discharge instructions in their preferred language

d. Other (please specify): ....

15) In your experience, how effective is the use of standardized discharge checklists?

a. Very effective

b. Effective

c. Neutral

d. Ineffective

e. Not sure

16) How do you measure the success of your discharge planning efforts?

a. Tracking readmission rates

b. Patient satisfaction surveys

c. Follow-up appointment attendance

d. Other (please specify): ....

17) What role does patient advocacy play in effective discharge planning?

a. Advocating for resources and support services

b. Ensuring patient understanding and compliance

c. Facilitating communication between healthcare providers

d. Other (please specify): ....

18) What challenges do you encounter most often during the discharge planning process?

a. Time constraints

b. Coordination among healthcare team members

c. Patient comprehension and readiness

d. Availability of resources (e.g., beds, equipment)

e. Other (please specify): ....

Section 4: Medication management:

19) How do you educate patients about potential side effects and adverse reactions?

a. Providing written information

b. Discussing side effects during medication reconciliation

c. Using patient-friendly language to explain risks

d. Other (please specify): ....

20) How do you monitor medication compliance post-discharge?

a. Follow-up phone calls or messages

b. Reviewing pharmacy refill records

c. Assessing patient understanding during follow-up appointments

d. Other (please specify): ....

21) What role does patient education play in improving medication adherence?

a. Empowering patients with knowledge about their medications

b. Enhancing patient communication with healthcare providers

c. Increasing patient confidence in managing their medications

d. Other (please specify): ....

22) How do you address patient concerns or questions about their medications?

a. Encouraging open dialogue during discharge planning

b. Providing contact information for medication-related queries

c. Offering medication education sessions

d. Other (please specify): ....

23) How do you assess the impact of medication management strategies on readmission rates?

a. Monitoring patient outcomes and health improvements

b. Tracking medication adherence rates

c. Analyzing readmission causes related to medication issues

d. Other (please specify): ....

24) What improvements would you suggest to enhance medication management post-discharge?

a. Increased access to pharmacist consultations

b. Better integration of medication data in electronic records

c. Enhanced patient medication education materials

d. Other (please specify): ....

Section 5: Patient education

25) Which topics do you prioritize in patient education to prevent readmissions? (Select all that apply)

a. Medication management

b. Signs and symptoms to watch for

c. Diet and nutrition guidelines

d. Exercise and physical activity recommendations

e. Pain management strategies

f. Other (please specify): ....

26) How do you communicate readmission risks to patients and their families?

a. Verbal explanations during discharge

b. Written materials

c. Interactive sessions with healthcare providers

d. Other (please specify)...

27) How do you address language barriers in patient education?

a. Providing interpreter services

b. Using language-appropriate written materials

c. Utilizing multilingual healthcare staff

d. Other (please specify): ....

28) In your opinion, what role does patient education play in preventing hospital readmissions?

a. Critical role

b. Important but not essential

c. Minimal role

d. Unsure

29) How do you assess the effectiveness of patient education efforts?

a. Patient feedback and comprehension checks

b. Monitoring patient adherence to instructions

c. Assessing patient outcomes and health improvements

d. Other (please specify): ....

30) What strategies do you use to reinforce patient education messages?

a. Follow-up phone calls

b. Sending reminder messages or emails

c. Scheduling additional education sessions

d. Other (please specify): ....

31) How do you collaborate with other departments or specialties within the hospital to address factors contributing to readmissions?

a. A. Regular multidisciplinary meetings

b. B. Shared patient care plans

c. C. Coordinated discharge planning

d. D. Other (please specify).....................................

Section 6: Follow-up and post-discharge care

32) How do you schedule follow-up appointments for discharged patients?

a. Providing specific appointment dates and times

b. Coordinating with primary care providers for scheduling

c. Utilizing reminder systems for patients

d. Other (please specify): ....

33) Which methods do you employ to ensure patients attend their follow-up appointments?

a. Conducting reminder calls or messages

b. Offering flexible scheduling options

c. Coordinating transportation assistance if needed

d. Other (please specify): ....

34) How do you assess the effectiveness of follow-up care in preventing readmissions?

a. Monitoring patient outcomes post-discharge

b. Assessing adherence to follow-up recommendations

c. Analysing readmission rates related to follow-up care

d. Other (please specify): ....

35) What role does patient compliance play in successful follow-up care?

a. Significant; it impacts patient outcomes and readmission risks

b. Moderate; it varies depending on patient circumstances

c. Minimal; other factors play a more critical role

d. Unsure/Varies

36) How do you handle missed follow-up appointments or non-compliance with care recommendations?

a. Conducting outreach to reschedule appointments

b. Providing patient education on the importance of follow-up care

c. Collaborating with social workers or case managers for support

d. Other (please specify): ....

37) How do you incorporate patient preferences and needs into follow-up care planning?

a. Offering choices in appointment times and locations

b. Considering patient transportation and accessibility issues

c. Discussing care options and goals with patients

d. Other (please specify): ....

Section 7: Current preventive practices

38) What strategies does your unit currently employ to prevent hospital readmissions? (Check all that apply)

a. Patient education

b. Discharge planning

c. Follow-up calls

d. Medication reconciliation

e. Home health referrals

f. Remote patient monitoring

g. Transitional care programs

h. Early warning systems

i. Other (please specify): ....

39) How effective do you find these strategies in reducing readmissions?

a. Very effective

b. Somewhat effective

c. Not effective

40) How do you ensure continuity of care between hospital discharge and outpatient services?

a. Electronic health record (EHR) integration

b. Direct communication with outpatient providers

c. Patient education on transition steps

d. Other (please specify): ....

41) How do you involve patients in the development of their discharge plan to prevent readmissions?

a. Shared decision-making processes

b. Patient and family meetings

c. Written discharge instructions with patient input

d. Other (please specify): ....

42) How do you ensure that patients understand their roles and responsibilities in preventing readmissions?

a. Verbal instructions during discharge

b. Written materials with clear instructions

c. Teach-back method

d. Other (please specify): ....

Section 8: Training and support

43) Have you received specific training related to preventing hospital readmissions?

a. Yes

b. No

44) How adequate do you think this training was in preparing you to prevent readmissions?

a. Very adequate

b. Somewhat adequate

c. Not adequate

45) Would additional training in readmission prevention be beneficial to your practice?

a. Yes

b. No

46) What specific areas of training would you find most valuable?

a. Patient communication skills

b. Utilization of discharge planning tools

c. Understanding of risk factors for readmission

d. Other (please specify): ....

47) How do you educate new staff members about the importance of readmission prevention?

a. Formal training sessions

b. Mentorship from experienced staff

c. On-the-job training

d. Other (please specify): ....

48) How do you stay updated on best practices and innovations in readmission prevention?

a. Professional conferences and workshops

b. Continuing education courses

c. Medical literature and journals

d. Other (please specify): ....

49) What resources or support do you need to enhance your ability to prevent readmissions?

....

Section 9: Patient engagement and education

50) How do you assess patients' understanding of their discharge instructions?

a. Verbal assessment

b. Written quizzes/forms

c. Teach-back method

d. Other (please specify): ....

51) What methods do you use to educate patients about their condition and discharge plan?

a. Verbal instructions

b. Printed materials

c. Video presentations

d. Other (please specify): ....

52) How do you tailor patient education to meet individual patient needs?

a. Assessing health literacy levels

b. Considering cultural and language preferences

c. Customizing content based on patient questions

d. Other (please specify): ....

53) How do you encourage patients to actively participate in their care post-discharge?

a. Setting specific goals with patients

b. Providing self-care instructions

c. Encouraging family involvement

d. Other (please specify): ....

54) How do you address language or cultural barriers in patient education to prevent readmissions?

a. Interpreter services

b. Multilingual educational materials

c. Cultural competency training for staff

d. Other (please specify): ....

55) How do you support patients in managing their medications post-discharge?

a. Medication reconciliation sessions

b. Providing pill organizers or reminders

c. Education on medication usage

d. Other (please specify): ....

56) How do you measure patient satisfaction with the discharge process and its impact on readmission rates?

a. Patient surveys

b. Feedback during follow-up calls

c. Focus groups with discharged patients

d. Other (please specify): ....

Section 10: Collaboration and communication

57) How would you rate the communication and collaboration between nursing staff and other healthcare providers in preventing readmissions?

a. Excellent

b. Good

c. Fair

d. Poor

58) How do you coordinate care transitions between different healthcare settings to prevent readmissions?

a. Standardized handoff protocols

b. Direct communication between providers

c. Patient health records sharing

d. Other (please specify): ....

59) How do you involve patients' primary care providers in their post-discharge care plan?

a. Sending discharge summaries

b. Scheduling follow-up appointments

c. Collaborative care planning meetings

d. Other (please specify): ....

60) How do you involve patients' families or caregivers in the readmission prevention process?

a. Family meetings during hospital stays

b. Educating families about discharge instructions

c. Providing caregiver support resources

d. Other (please specify): ....

61) How frequently do you utilize resources such as social workers, case managers, or community resources to prevent readmissions?

a. Very frequently

b. Occasionally

c. Rarely

d. Never

What are your suggestions to reduce readmission?

……
……
……
……
